# Sleep, Mental Health, and Cognition Across Diverse Adult Populations

**DOI:** 10.1093/geroni/igad104.1282

**Published:** 2023-12-21

**Authors:** Soomi Lee, Christopher Kaufmann, Adam Spira

## Abstract

Sleep is often impaired in late life, raising concerns about the effect of poor sleep on mental and cognitive health in aging individuals. This symposium brings together five rigorous studies that show how poor sleep may be an under-recognized risk factor for mental health and cognitive outcomes in the second half of life. Specifically, this symposium showcases data from a variety of study cohorts (including Midlife in the United Study, Einstein Aging Study, and Study of Muscle, Mobility, and Aging), using diverse indicators of subjective and objective sleep. Paper 1 focuses on healthcare workers (HCWs), a group critical for the delivery of quality patient care, and examines both individual and joint associations of poor sleep and pain with mental health in HCWs vs. non-HCWs. Paper 2 investigates the association between EEG-measured sleep and depressive symptoms in older adults. Paper 3 explores the relationship between intra-individual variability of sleep and mild cognitive impairment (MCI) and identifies gender differences in these associations. Paper 4 establishes the associations of actigraphy-measured sleep and circadian rest-activity rhythms with multiple domains of cognition in older adults. Paper 5 examines the effect of latent dose/duration patterns of common sleep medications on development of Alzheimer’s disease and related dementias. The discussant, Dr. Adam Spira, will integrate key findings from these studies, discuss their contributions to the literature, and consider opportunities for future research. This is a Sleep, Circadian Rhythms and Aging Interest Group Sponsored Symposium. This is a collaborative symposium between the Alzheimer’s Disease and Related Dementias, Geroscience, and Health Behavior Change Interest Groups.

